# Effect of Temperature on Microstructure and Mechanical Properties of Fe-9Ni-2Cu Steel during the Tempering Process

**DOI:** 10.3390/ma14237141

**Published:** 2021-11-24

**Authors:** Xi Huang, Lianbo Wang, Zemin Wang, Zhanyong Wang, Qingdong Liu

**Affiliations:** 1 School of Materials Science and Engineering, Shanghai Institute of Technology, Shanghai 201418, China; huangxi97@foxmail.com (X.H.); wanglianbo021@hotmail.com (L.W.); 2 Collaborative Innovation Center for Advanced Ship and Deep-Sea Exploration, Shanghai Jiaotong University, Shanghai 200240, China; qdliu@sjtu.edu.cn

**Keywords:** steel, tempering, reversed austenite, fresh martensite, temperature

## Abstract

In this paper, scanning electron microscopy (SEM), transmission electron microscopy (TEM), X-ray diffraction (XRD), X-ray stress meter (XRSA), atom probe tomography (APT), hardness, and tensile tests were used to study the effect of tempering temperature on the microstructure and properties of Fe-9Ni-2Cu steel. The results show that after the quenched samples were tempered at 460 °C for 2 h, the hardness values increased from 373 to 397 HV, and elongation also increased from 13% to 16%. With the tempering temperature increasing from 460 to 660 °C, the hardness firstly decreases from 397 to 353 HV and then increases to 377 HV, while the elongation increases to 17% and then decreases to 11%. The variation of the mechanical properties greatly depends on the evolution of the Cu-rich phase and carbides. The precipitation strengthening of the Cu-rich phase and carbides leads to the increase of hardness, but when the precipitate is coarsened, the precipitation strengthening weakens, and then, the hardness increases. When the tempering temperature is 560 °C, a large amount of stable reverse transformation austenite was formed with a content of 7.1%, while the tensile strength reached the lowest value of 1022 MPa and the elongation reached the maximum value of 17%.

## 1. Introduction

The development of the global new energy industry, the adjustment of the domestic energy structure, and the increase of environmental protection pressure have led to a sharp increase in the demand for natural gas, so it is necessary to increase the quality of vehicles for natural gas. 9Ni steel with high strength and high low-temperature toughness has attracted a great deal of attention over the past few years [1,2,3,4,5,6].

At present, the research on 9Ni steels with excellent properties mainly focuses on the contents and stability of the reversed variant austenite obtained by QT (Quenching + Tempering) and QLT (Quenching + Lamellarizing + Tempering) processes [7,8,9,10], achieving excellent comprehensive mechanical properties of strength and toughness. The toughness is better after QLT treatment, but the strength decreases more. Rongbin Li et al. [11] obtained good mechanical properties through QLT, with tensile strength reaching 734.85 MPa and elongation reaching 27.58%. The low-temperature toughness and elongation of the samples treated by the QLT process are improved, but the strength is still relatively low [12]. Wei Hou et al. [13] modified the diffusion of alloying elements by increasing the duration of heat treatment in the critical. After six cycles, the tensile strength was successfully increased from 923 to 1001 MPa, and the elongation was 14.8%. However, the QLT process is complex and the cost is high, which is not conducive to wide industrial production. The traditional QT (Quenching + Tempering) process is more convenient and conducive to large-scale industrial production [14]. Unfortunately, the steel sheet heat-treated by the QT process still cannot guarantee safety during service at low temperatures due to the lack of enough toughness. How to further enhance toughness at low temperature without reducing strength still has strong research significance.

In this paper, the tailored QT process is applied to obtain good mechanical properties at low temperatures. Results have shown that the content and stability of the reversed austenite are important indexes of the properties of 9Ni steel. Reverse austenite is formed by the accumulation of alloying elements, which leads to the transformation of supersaturated solid solution at lower temperatures below Ac1. It contains higher C, Ni, Mn, and other impurity elements, and it remains stable at low temperature [15]. In the QT process, the tempering temperature is the main factor controlling element distribution A kind of steel (Fe-9Ni-2Cu) for an LNG (Liquefied Natural Gas) storage tank containing Ni and Cu elements is introduced in this paper. The effects of tempering temperature on the microstructure and properties of Fe-9Ni-2Cu steel were investigated by room temperature tensile, XRD, TEM, and APT.

## 2. Experiment

The steel used in the experiment is a hot-rolled steel plate with a thickness of 25 mm produced by the steel mill. The chemical composition is shown in Table 1. According to the composition characteristics, it is referred to as Fe-9Ni-2Cu steel. The steel plate was wire-cut into samples and held at 900 °C for 1 h before water quenching. Then, the samples were tempered for 2 h at 460 °C, 510 °C, 560 °C, 610 °C, and 660 °C, respectively.

**Figure 1:**
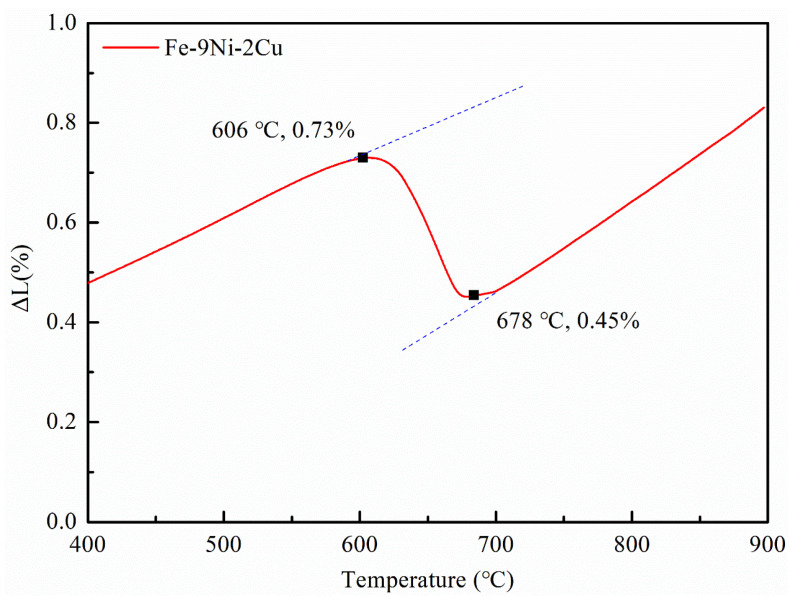
Phase transformation temperature of Fe-9Ni-2Cu steel measured by thermal expansion methods.

The thermal expansion curve of steel was measured by the German Netzsch thermal dilatometer, as shown in Figure 1. It can be seen that the austenitic transition start temperature (Ac1) and finishing temperature (Ac3) were about 606 °C and 678 °C, respectively (the heating rate is 5 °C/min).

The content of reversed austenite in steel samples was determined using a D/Max 2200PC X-ray diffractometer. The diffraction peaks of (200) α, (211) α, (200) γ, (220) γ, and (311) γ were selected to measure the diffraction angles and integral intensity, and the volume fraction of reversed austenite was calculated [16]. The content and stability of reversed austenite samples stored in liquid nitrogen at room temperature and −196 °C for 30 min were determined and analyzed by using a proto-LXRD X-ray apparatus from Canada.

After grinding and polishing, the sample was eroded by 4% nitric acid alcohol solution, and the microstructure and tensile fracture morphology were observed by a SANS-CMT5105 Optical microscope, FEI Quanta200 FEG scanning electron microscope, and JEM-2100F thermal field emission transmission electron microscope. TEM samples were cut into 0.20 mm slices and polished to about 50 μm. After thinning at −40 °C, TEM experiments were conducted. Atom probe chromatography was used to analyze the formations of atomic clusters during tempering at different temperatures. The needle sample was prepared as 0.5 mm × 0.5 mm × 20 mm by two-step electropolishing [17].

**Figure 2:**
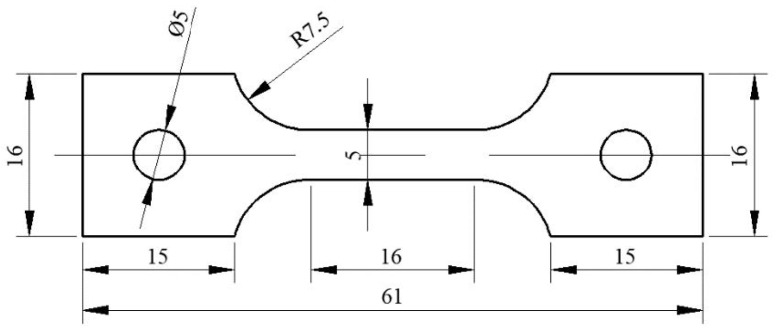
Dimensional drawing of tensile specimen (units: mm).

Hardness was measured by a 402SXV Vickers microhardness tester with an applied load of 200 g for 5 s. Each sample was measured seven times, and the average values were extracted and used for analysis. Tensile specimens were prepared by sampling in the direction perpendicular to rolling, and the testing standard was ATSM E8/E8M. The size of the tensile sample with a thickness of 1.5 mm is shown in Figure 2. A Zwick/Roell Z100 universal material testing machine was used for a normal temperature tensile test, and each group of samples was tested third in parallel.

## 3. Results

### 3.1. Mechanical Properties

**Figure 3:**
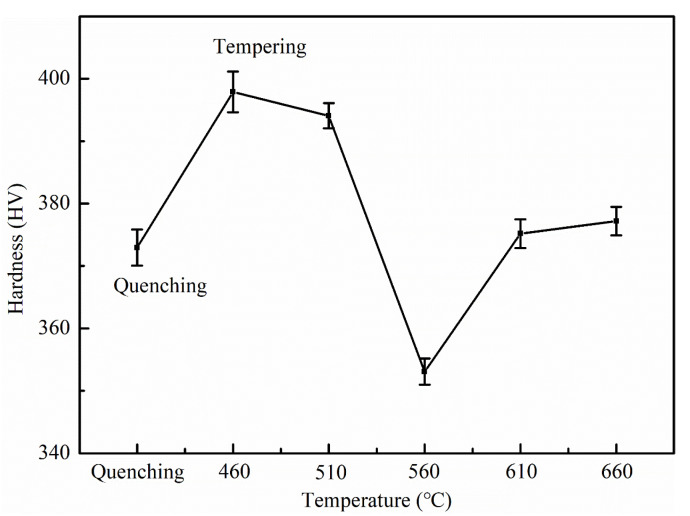
Hardness changes of Fe-9Ni-2Cu steel with the increase of temperatures.

Figure 3 shows the distribution of hardness of Fe-9Ni-2Cu steel after quenching and tempering at different temperatures. As shown in Figure 3, the tempering temperature has a significant impact on the hardness of Fe-9Ni-2Cu steel. In comparison with quenched Fe-9Ni-2Cu steel, an increase of hardness is observable for the tempered Fe-9Ni-2Cu steel at 460 °C. With the tempering temperature further increasing from 460 to 660 °C, the hardness firstly decreases from 397 to 353 HV and then increases to 377 HV.

**Figure 4:**
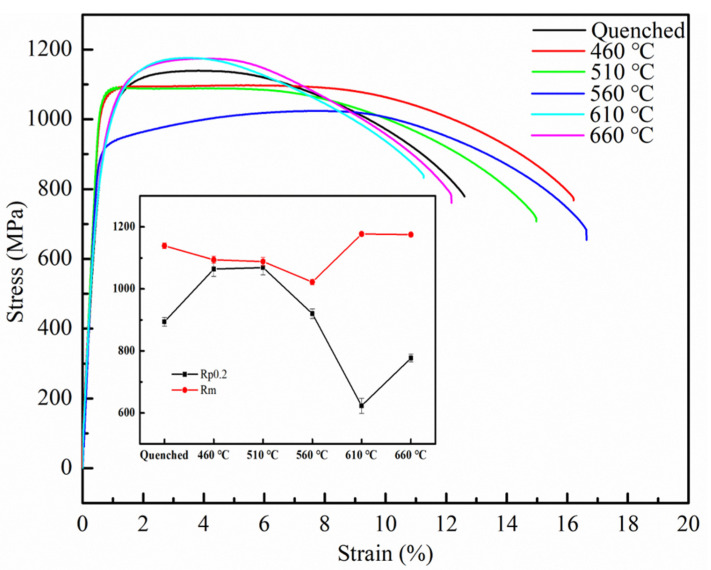
Tensile property changes of Fe-9Ni-2Cu steel with the increase of temperatures.

Figure 4 shows the tensile stress–strain curves of samples tempered at different temperatures at room temperature. It can be seen from Figure 4 that the yield strength and tensile strength of the quenched sample are 894 MPa and 1139 MPa, respectively. After tempering, the elongation of the sample shows a trend of first increasing and then decreasing. It can be seen from the figure that when tempering at 460 °C and 510 °C, the strength of the sample steel is in a platform [13]. The strength reaction is more accurate than hardness, and considering the error value of hardness measurements, the two confirm each other; this may be due to the combination of Cu-rich phase and carbides. At low tempering temperature, the elongation and yield strength of the samples increased significantly, while the yield strength decreased rapidly, and the tensile strength increased rapidly when the tempering temperature continued to increase. When tempered at 560 °C, the tensile strength of the sample steel is 1022 MPa, and the elongation reaches 17%.

**Figure 5:**
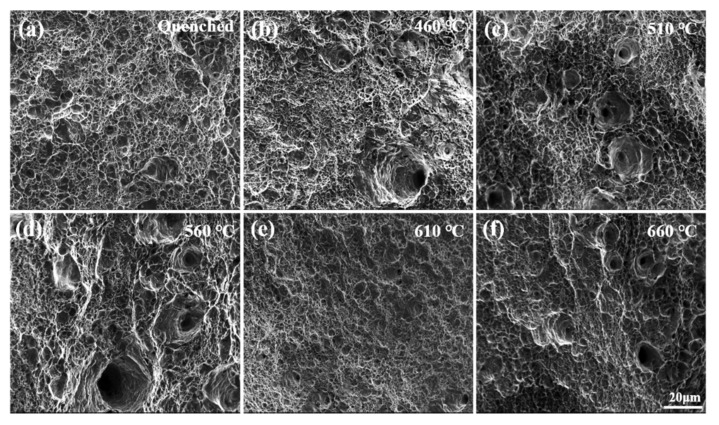
Tensile fracture micromorphology images of the Fe-9Ni-2Cu steel with the increase of temperatures. (**a**) Quenched; (**b**) 460 °C; (**c**) 510 °C; (**d**) 560 °C; (**e**) 610 °C; (**f**) 660 °C.

Figure 5 shows the tensile fracture morphology of the samples after tempering at different temperatures. It can be seen from the fracture morphology that the samples all present ductile fracture. It can be seen from Figure 5b that the number of dimples after tempering gradually increases, compared with the quenched samples, and the toughness and elongation are improved. When the temperature increases to 510 °C, the dimple depth decreases, and the elongation decreases to a certain extent. When the temperature rises to 560 °C, the number of dimples increases again, and the dimples become larger and deeper, and the elongation of samples increases. In Figure 5e, the size of the dimple decreased significantly, and the fracture morphology showed toughness + quasi cleavage fracture. The toughness of the sample steel decreased significantly. As the tempering temperature increases further, the dimples in Figure 5f increase, but the increase in toughness and elongation is not obvious [18]. It indicates that the fresh martensite transformed from unstable reversed austenite during the cooling process damages the toughness and elongation greatly.

### 3.2. Microstructure Characterization

**Figure 6:**
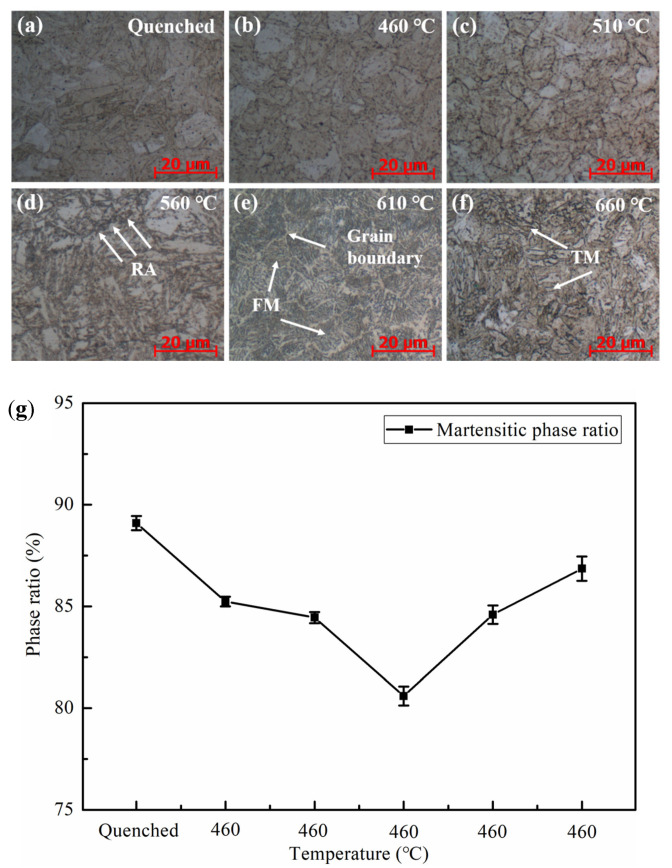
OM images of the Fe-9Ni-2Cu steel with the increase of temperatures. (**a**) Quenched; (**b**) 460 °C; (**c**) 510 °C; (**d**) 560 °C; (**e**) 610 °C; (**f**) 660 °C; (**g**) phase ratio.

Figure 6 shows the microstructure of Fe-9Ni-2Cu steel after quenching and tempering at different temperatures. Figure 6 shows that the microstructure of Fe-9Ni-2Cu steel changes significantly with the increase of tempering temperature. As shown in Figure 6a, the main structures after quenching are lath bainite. Figure 6b,c show the metallographic structure of the sample at 460 °C and 510 °C, respectively. Lath martensite and retained austenite are the main structures in the matrix. As shown in Figure 6d, when the temperature increases up to 560 °C, the lath martensite matrix is relatively pure, and a large amount of reversed austenite is produced at the grain boundary as indicated by the white arrow in the figure. As can be seen in Figure 6e, with the increase of temperature, white bands appear in the boundary part, while dense lath structures form inside martensite, which is fresh martensite. It can be seen from Figure 6f that tempered martensite is the main structure obtained during tempering at a higher temperature in the two-phase region. Figure 6f shows the proportion of the martensite phase. It can be seen from Figure 6g that when the tempering temperature rises from 460 to 660 °C, the proportion of the martensite phase decreases from 89.13% to 80.59% and then increases to 86.85%.

**Figure 7:**
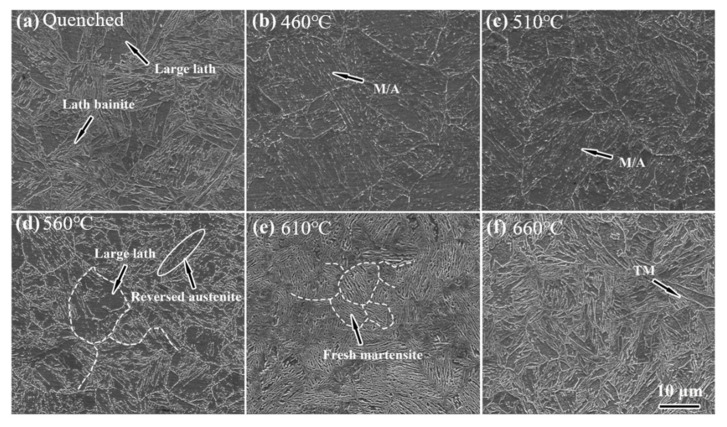
SEM images of the Fe-9Ni-2Cu steel with the increase of temperatures. (**a**) Quenched; (**b**) 460 °C; (**c**) 510 °C; (**d**) 560 °C; (**e**) 610 °C; (**f**) 660 °C.

Figure 7 shows SEM images of Fe-9Ni-2Cu steel after quenching and tempering at different temperatures, using the secondary electron mode. It can be seen that the quenched microstructure of the steel is lath bainite (see in Figure 7a). Figure 7b,c show the scanning microstructure of samples at 460 °C and 510 °C. When the single-phase region is tempered, the matrix is characterized by lath martensite and contains martensite/austenite (M/A) mixtures rich in C and Ni elements. With the increase of tempering temperature, bright dotted linings appear gradually in the matrix of lath martensite, and bright linings appear at the grain boundary of lath martensite at 560 °C, forming reverse austenite with a high C content. When the tempering temperature reaches 610 °C, the mixture of reversed austenite and fresh martensite is formed [13]. When the tempering temperature rises to 660 °C, it enters the upper part of the temperature of the coexistence region. At this time, the reversed austenite generated has higher content and worse stability and forms secondary martensite after cooling down.

**Figure 8:**
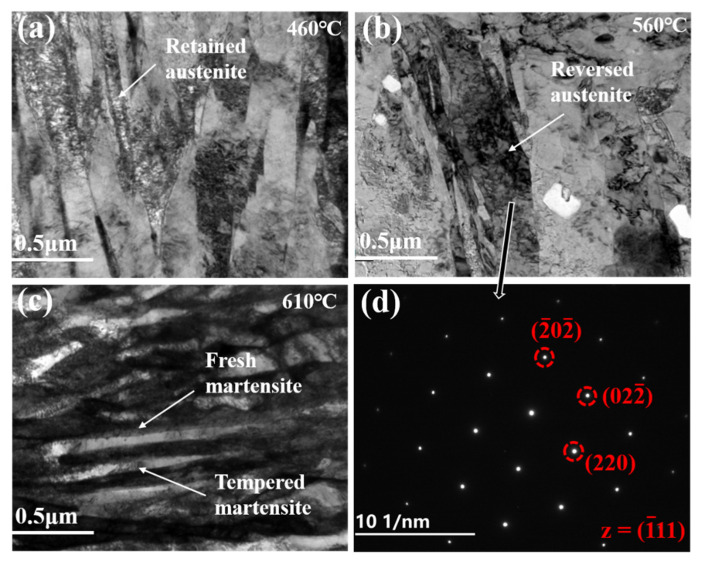
TEM images of the Fe-9Ni-2Cu steel at different tempering temperatures. (**a**) 460 °C; (**b**) 560 °C; (**c**) 610 °C; (**d**) diffraction spot of 560 °C.

Figure 8 shows the TEM morphology of the samples after tempering at different temperatures. Figure 8a shows that the Fe-9Ni-2Cu steel after QT treatment under 460 °C possesses lath martensite and a small amount of retained austenite (needle-like). As the sample is QT under 560 °C, reversed austenite (thin-film) forms near the lath martensite grain boundary. Further increasing the QT temperature, as shown in Figure 8c, alternating light and dark martensite lath bundles appear. Sun also found that the microstructure was composed of long and straight lath martensite with a high density of dislocations in the lath when quenching in the coexistence region, which is in accordance with the result in the current work [19].

**Figure 9:**
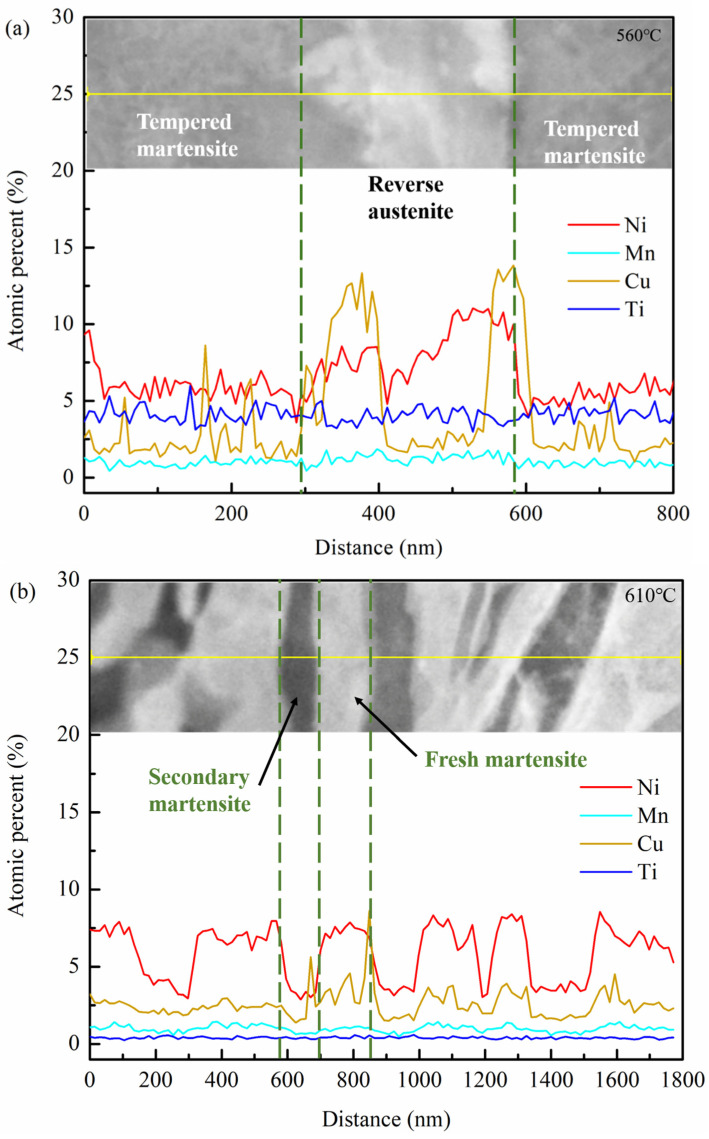
STEM image analysis after tempering at different temperatures. (**a**) 560 °C; (**b**) 610 °C.

Figure 9 shows STEM image analysis after tempering at different temperatures. It can be seen from Figure 9a that Ni and Cu elements are enriched in the reversed austenite region, and Cu elements’ segregation occurs at the junction of reversed austenite and lath martensite. Figure 9b shows TEM energy spectrum analysis after tempering at 610 °C. The contents of Ni, Cu, and Ti elements in the fresh martensite decrease, Ni elements segregate in the fresh martensite, while Cu elements mainly segregate at the boundary of fresh martensite.

**Figure 10:**
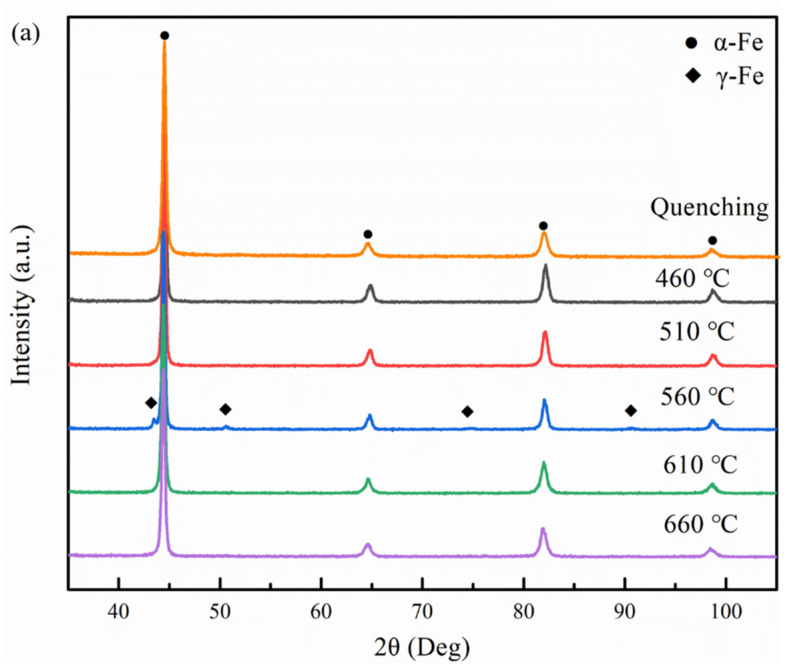

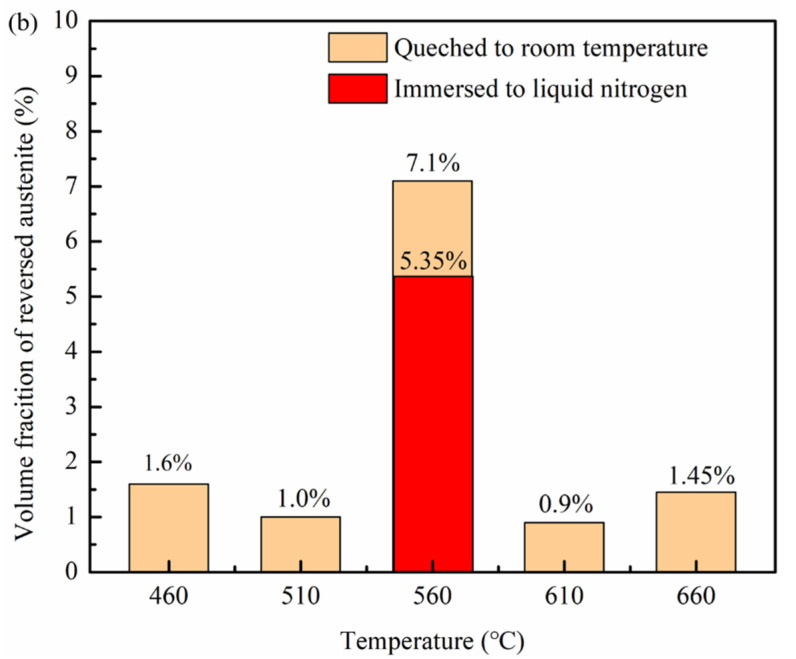
Austenite content and stability of Fe-9Ni-2Cu steel are reversed after quenching and tempering at different temperatures. (**a**) XRD; (**b**) low-temperature stability.

Figure 10 shows XRD patterns of Fe-9Ni-2Cu steel after quenching and tempering at different temperatures. As can be seen from Figure 10a, only the α-Fe characteristic peak can be seen after quenching and tempering at 460 °C, but no significant γ-Fe characteristic peak can be seen [20]. When the tempering temperature increases to 510 °C, only the weak γ-Fe characteristic peak can be seen at 2θ = 43.60°. When the tempering temperature rises to 560 °C, the XRD curve shows a significant γ-Fe characteristic peak, which indicates that there is more reversed austenite in the sample steel at this time. With the further increase in tempering temperature, the γ-Fe characteristic peak disappears. Figure 10b shows the stability test of reversed austenite. The content of reversed austenite after quenching and tempering samples at different temperatures was stored at room temperature and −196 °C liquid nitrogen for 30 min. As can be seen from the figure, with the increase of tempering temperature, the reversed austenite content in the sample steel shows an overall trend of first increasing and then decreasing, and it reaches a maximum value of 7.1% at 560 °C. After low-temperature treatment, the reversed austenite content is still 5.35%. The stability of austenite decreases with temperature increase [21], and the content decreases with cooling.

**Figure 11:**
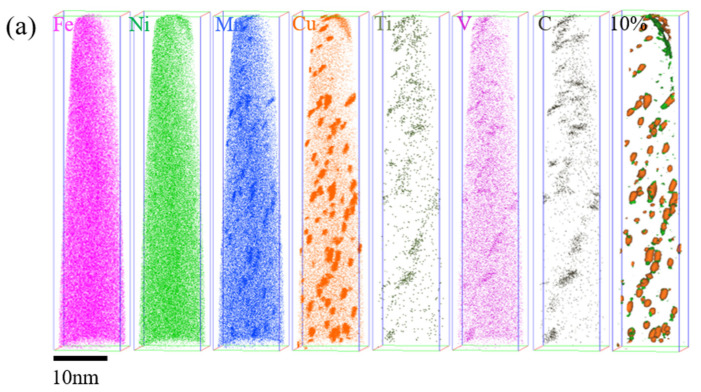

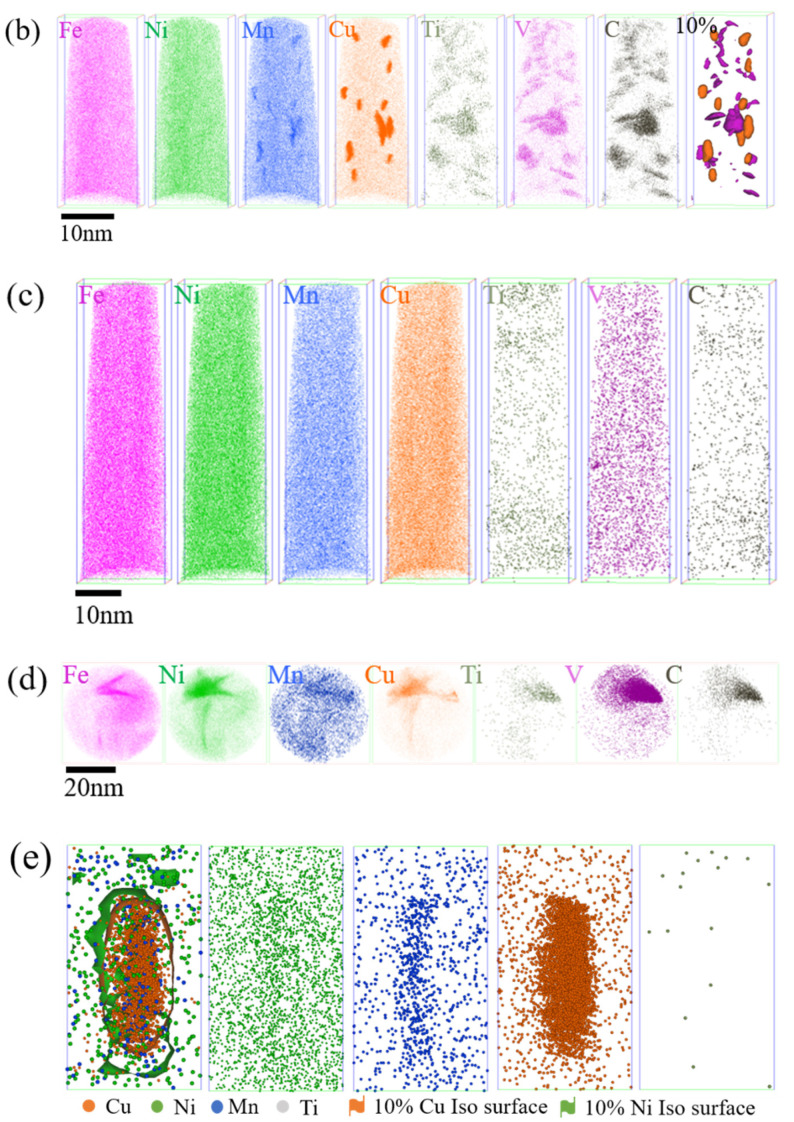
Spatial distribution of elements in Fe-9Ni-2Cu steels after tempering at different temperatures. (**a**) 460 °C; (**b**) 560 °C; (**c**) 610 °C; (**d**) z-610 °C; (**e**) 10% Cu + Ni + Mn Iso surface after tempering at 560 °C.

Figure 11 shows the spatial distribution of elements in Fe-9Ni-2Cu steel after tempering at different temperatures. Figure 11a shows the APT analysis diagram at 460 °C tempering. The distribution of Mn, Cu, Ti, V, and C atoms are segregated at 460 °C tempering. Cu atoms tend to agglomerate in the supersaturated matrix and form high-density metastable Cu-rich atomic clusters. When the tempering temperature is 560 °C, the Cu-rich phase and carbides are coarsened, and the size of the Cu-rich phase increases, and the number of Cu-rich phases decreases [22]. As shown in Figure 11, when the tempering temperature is 560 °C, NiMn tends to be clustered at edge of the Cu-rich phase. When the tempering temperature is further increased to 610 °C, after the decomposition of the Cu-rich phase and carbides, Cu atoms are re-dispersed into the matrix. It can be seen from the *X*-axis that all elements are evenly distributed, but different degrees of segregation appear from the *Z*-axis.

## 4. Discussion

With the increase of temperature, the microstructure of the sample steel presents a transition from lath martensite to reversed austenite to fresh martensite to secondary martensite.

When at low tempering temperature in the single-phase region, the kinetic energy for the diffusion of the alloying elements is insufficient, and the matrix consists of lath martensite and a small amount of retained austenite, as shown in Figure 7. As for the quenched sample tempered at 460 °C, the main structures in matrix are tempered martensite and needle-like retained austenite (see Figure 8). The presence of the clustered Cu-rich phase leads to the increased hardness, and the increase of dimples on the tensile fracture surface illustrates the enhanced toughness. As discussed in Figure 7c above, the M/A mixtures increase, the strength and hardness remain unchanged, and the elongation decreases. Köse [23] has also declared that the formation of carbide in the weld duplex stainless steels leads to a decrease of strength and a decrease in elongation. With the increase of tempering temperature, APT analysis shows that the Cu-rich phase and carbides are gradually coarsened, and the precipitation-strengthening effect is weakened, leading to the decrease of strength and hardness. Liu [17] confirmed that the Ni segregation is beneficial to the rapid growth of the Cu-rich phase at the interface. Bagheri et al. [24] proposed that the size of precipitated phases such as M/A increases with the increase of tempering temperature, leading to the decrease of hardness. After tempering at 560 °C, which approaches Ac1 temperature, the diffusion ability of alloying elements is enhanced, and the needle-like retained austenite is transformed to reversed austenite (see Figure 8). At the same time, the content of reversed austenite increases, and its stability increases as well, and 5.35% of austenite remains after cryogenic treatment. Divya Jain et al. [25] confirmed that the segregation of alloying elements can improve the content and stability of reversed austenite. The stable reversed austenite can effectively enhance the toughness of the sample. Araujio et al. [26] have pointed out that the thin film austenite allows the flowing of matter along with interfaces by a lubrication effect and improves the toughness of the material effectively. As shown in Figure 9a, Ni, Mn, Cu, and other elements tend to be concentrated in and near the boundary of reversed austenite, and the alloying elements adsorbed inside enhance the stability of reversed austenite, and the stable reversed austenite in the matrix significantly improves the toughness of steel. Wang et al. [27] confirmed that thin-film austenite has a larger specific surface area than granular austenite and has greater resistance induced by interfacial energy and better toughness during its transformation to martensite.

When the tempering temperature rises to the coexistence region, the hardness of the steel increases again, while the toughness decreases. After tempering at 610 °C, fresh martensite and tempered martensite with alternating distribution, as shown in Figure 8c, are formed. In the heating process, more reversed austenite is generated, but the content of absorbed alloying elements decreases, as shown in Figure 9b. The stability of the reverse austenite declines; thus, it transform into fresh martensite with rich elements of C, Ni, and Mn [28]. The decrease of reversed austenite and the appearance of fresh martensite are greatly responsible for the increase of steel strength and hardness. When the tempering temperature rises to 660 °C, it approached the critical temperature of Ac3, the alloying elements were fully diffused, only tempered martensite emerged, as shown in Figure 7. Thereby, the toughness and strength of the sample steel were improved.

## 5. Conclusions

(1) The tempering temperature has a significant effect on the content of reversed austenite. With the increase of tempering temperature, the content of reversed austenite increases first and then decreases. The reversed austenite nucleated at the boundary of lath martensite first, and the higher content and better stability of the reversed austenite could be obtained after tempering at 560 °C.
(2) After tempering, the toughness and elongation of Fe-9Ni-2Cu steel are significantly improved. The stable reversed austenite can adsorb alloying elements in the matrix, which can purify the matrix to form large strip bundles and improve toughness greatly. The hardness of fresh martensite is improved to some extent.
(3) After tempering Fe-9Ni-2Cu steel at different temperatures, Ni and Mn atoms tend to converge near the Cu atomic clusters to form a Cu-rich phase at 460 °C, which has an obvious precipitation-strengthening effect on the strength and hardness of the sample steel. As shown in Figure 11, when the tempering temperature is 560 °C, NiMn tends to be clustered at edge of the Cu-rich phase. At 610 °C, the Cu-rich phase and carbides are re-dispersed, and Cu atom segregation occurs at the reversed austenite/matrix interface.

## Figures and Tables

**Table 1:** Chemical analysis of experimental steel (mass fraction, %).

Element	C	Si	Mn	Ni	Cu	V	Ti	Al	Fe
Mass fraction (%)	0.057	0.070	0.59	9.0	2.0	1.5	0.011	0.02	Ball

## Data Availability

No new data were created or analyzed in this study. Data sharing is not applicable to this article.
